# Echolocation by the harbour porpoise: life in coastal waters

**DOI:** 10.3389/fphys.2013.00052

**Published:** 2013-04-15

**Authors:** Lee A. Miller, Magnus Wahlberg

**Affiliations:** ^1^Institute of Biology, University of Southern DenmarkOdense, Denmark; ^2^Fjord&BæltKerteminde, Denmark

**Keywords:** echolocation, biosonar, hearing, harbor porpoise, *Phocoena phocoena*, noise, clutter, coastal waters

## Abstract

The harbor porpoise is one of the smallest and most widely spread of all toothed whales. They are found abundantly in coastal waters all around the northern hemisphere. They are among the 11 species known to use high frequency sonar of relative narrow bandwidth. Their narrow biosonar beam helps isolate echoes from prey among those from unwanted items and noise. Obtaining echoes from small objects like net mesh, net floats, and small prey is facilitated by the very high peak frequency around 130 kHz with a wavelength of about 12 mm. We argue that such echolocation signals and narrow band auditory filters give the harbor porpoise a selective advantage in a coastal environment. Predation by killer whales and a minimum noise region in the ocean around 130 kHz may have provided selection pressures for using narrow bandwidth high frequency biosonar signals.

## INTRODUCTION

The harbor porpoise, *Phocoena phocoena*, is a small whale about 1.5 m long and weighing about 65 kg. The species has a large distribution and ranges as far south as Mauretania and as far north as western Greenland and northern Alaska (Culik, 2011). Harbor porpoises seem to prefer coastal waters, even though they are sometimes seen in the middle of the ocean (Haug et al., 2003; MW, personal observation).

Like other toothed whales, harbor porpoises use echolocation to hunt for their prey, such as fish and squid. They emit intense ultrasonic signals in a narrow sound beam and listen for echoes (Busnel and Dziedzic, 1967; Møhl and Andersen, 1973; Miller, 2010; Koblitz et al., 2012). Their signals are narrow in bandwidth and high in frequency (NBHF; Au, 1997). They share this type of signal with at least three of the other six species in the porpoise family Phocoenidae, the four species of *Cephalorhynchus *dolphins, two species of southern ocean *Lagenorhynchus* dolphins, and the Franciscana dolphin, *Pontoporia blainvillei* (Morisaka and Connor, 2007; Kyhn et al., 2009, 2010; Tougaard and Kyhn, 2010; Melcón et al., 2012). All of the species listed are found in coastal habitats, but also pelagic. The only truly pelagic species of toothed whales known to use NBHF clicks is the pygmy sperm whale, *Kogia breviceps* (Madsen et al., 2005).

From phylogeny (Steeman et al., 2009), one would expect the broadband click to be the ancestral odontocete biosonar signal. What selective pressures caused the appearance of NBHF signals in a few primarily coastal odontocetes? Previously suggested answers to this question have focused on acoustic mechanisms like extracting an echo from noise and antipredator behavior (Andersen and Amundin, 1976; Madsen et al., 2005; Morisaka and Connor, 2007). Here we review such mechanisms in light of new data gathered on noise sources and the acoustic behavior, hearing and sound production of harbor porpoises.

## ECHOLOCATION BEHAVIOR OF HARBOR PORPOISES

**FIGURE 1:**
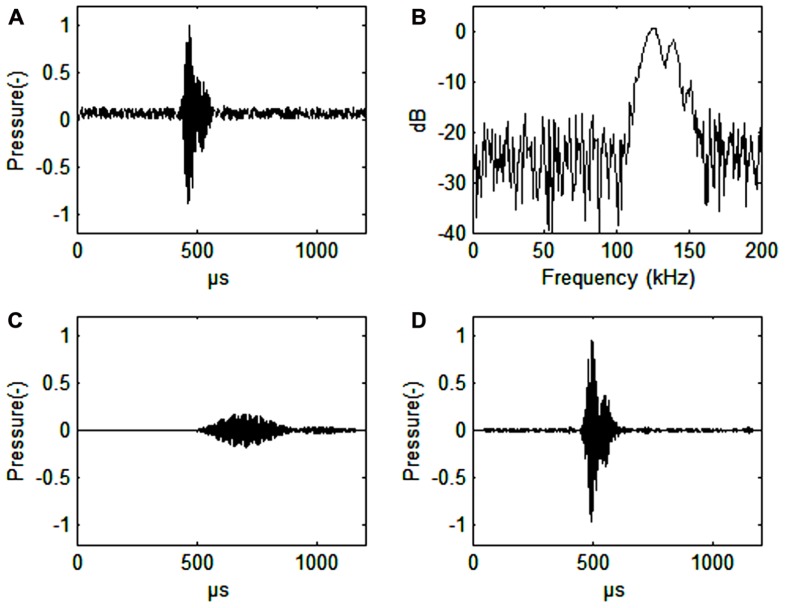
**(A)** Harbor porpoise click. Signal-to noise ratio 16 dB. **(B)** Spectrum of a harbor porpois click. **(C)** Harbor porpoise click filtered with a band pass filter (128–132 kHz, 4th order Butterworth filter), or about the width of the frequency auditory filter (as estimated by Popov et al., 2006). The axes for pressure are in relative, but comparable values. The dB values are also relative. **(D)** Harbor porpoise click filtered through a simulated third-octave band filter (center frequency 130 kHz, bandwidth 30 kHz, 4th order Butterworth filter). Note the vastly improved time resolution that is only about 10 μs delayed relative to the timing of the original signal **(A)**. Also note the improved signal to noise ratio (34 dB) in C relative to the third-octave filtered signal (30 dB) in D.

Harbor porpoise clicks are centered between 130 and 140 kHz with a bandwidth of 6–26 kHz (Dubrovskij et al., 1971; Møhl and Andersen, 1973; Villadsgaard et al., 2007; **Figure 1B**). The duration of the click is around 44–113 μs (Villadsgaard et al., 2007; **Figure 1A**). The signals are produced in the nasal passages just below the blowhole and emitted through the melon in a narrow 11–13° beam (Koblitz et al., 2012; Kyhn et al. submitted, see acknowledgments). The phonic lips, air sacs, and the melon are all involved in sound production (Madsen et al., 2010; Miller, 2010).

Like other toothed whales, harbor porpoises adjust the inter-click intervals of their sound emissions so that the echo does not overlap with the next click emission (Akamatsu et al., 2007; Verfuss et al., 2009; Wisniewska et al., 2012). While searching for prey the normal inter-click interval is around 30–100 ms. As the animal approaches the prey, the inter-click intervals become progressively shorter and ends in a “buzz,” with click intervals of about 1.5 ms when the porpoise is about a meter or two from the prey (Verfuss et al., 2009). The porpoise reduces the amplitude of its clicks by approximately 6 dB per halved distance to the target (Atém et al., 2009; Linnenschmidt et al., 2012a,b; Wisniewska et al., 2012).

The audiogram of the harbor porpoise has one of the widest bandwidths of any animal. The best sensitivity is found between about 80 and 140 kHz (Kastelein et al., 2002, 2010). Harbor porpoises can adjust their hearing when listening for echoes at various distances. That is, when the test target is moved toward the animal, the hearing sensitivity and the level of its biosonar signal are progressively decreasing so that the neural response of the echo stays at about the same level (Linnenschmidt et al., 2012a). In this way, the perceived echo level can be adjusted to a convenient amplitude within the dynamic range of the neuro-auditory system.

After transmitting the intense, ultrasonic pulses, harbor porpoises listen for the faint echoes returning from fish and other items in the water. Besides receiving the signal, ambient noise is also picked up by the hearing system. The porpoise has several ways to reduce the amount of received noise. First, the hearing system is directional, so that most energy is picked up in a cone 22° wide in front of the animal (Kastelein et al., 2005). Thus, the directionality of the receiving system is about twice as wide as that of the transmission system (22° rel. 11–13°). Secondly, when listening to an echo only noise within a restricted bandwidth will disturb perception of that echo. In humans, this bandwidth, called the critical bandwidth, is approximately 23% of the center frequency in question for higher frequencies. These are the so-called third octave bands that form a series of constant Q (quality) filters. Third octave bands are also known to approximately describe some of the critical bands in the auditory systems of dolphins and other odontocetes at higher frequencies (Au and Moore, 1990; Au, 1993).

Popov et al. (2006) used tonal masking to describe the auditory filter functions of a harbor porpoise and Kastelein et al. (2009) measured the critical ratio (which is an estimate of the critical band) of two harbor porpoises using a psychophysical paradigm. Popov et al. (2006) found that the critical bands in the frequency range of echolocation are 3–4 kHz wide. On the other hand, Kastelein et al. (2009) measured critical ratios of 34 and 37 dB at these frequencies indicating a bandwidth of 2.5–5.0 kHz using Fletcher’s assumption (Fletcher, 1940). Differences in experimental design could explain the discrepancy between these measurements, as one study used a tonal masker and auditory brainstem recordings and the other used psychophysics for tonal detection in narrow-band noise.

An interesting feature of both of these estimates is that the critical bands do *not* always seem to be a linear function of the center frequency at the frequency band of echolocation, which is the most common feature of critical bands for almost all other vertebrates (Fay, 1988). Instead, porpoises seem to have rather constant auditory filter bandwidths at echolocation frequencies. This has recently been supported by data from the bottlenose dolphin, *Tursiops truncatus*, (Lemonds et al., 2012). The frequency bands measured from both species are narrower than the actual bandwidth of the echolocation signals. There are currently no data available to understand why such filters are advantageous during echolocation.

A narrow band auditory filter gives poor time resolution (**Figure 1C**), which an odontocete needs for determining distance to prey. From observations on blindfolded individuals it is quite obvious, however, that the harbor porpoise knows exactly were the fish is during prey capture (Miller, 2010). Wider filters will improve time resolution (**Figure 1D**). We predict that the harbor porpoise has narrow band and wide band auditory filters running in parallel to effectively extract echoes from noise without losing time resolution. This seems to be the case for the bottlenose dolphin, where wide band auditory filters (constant Q) are found up to 100 kHz in parallel with constant bandwidth filters (about 10 kHz) at auditory frequencies from 60 to 100 kHz (Lemonds et al., 2012). The wide band auditory filters provide good temporal resolution while the narrow band auditory filters may be better for discriminating between echoes of various origins.

## NOISE IN THE COASTAL ENVIRONMENT

**FIGURE 2:**
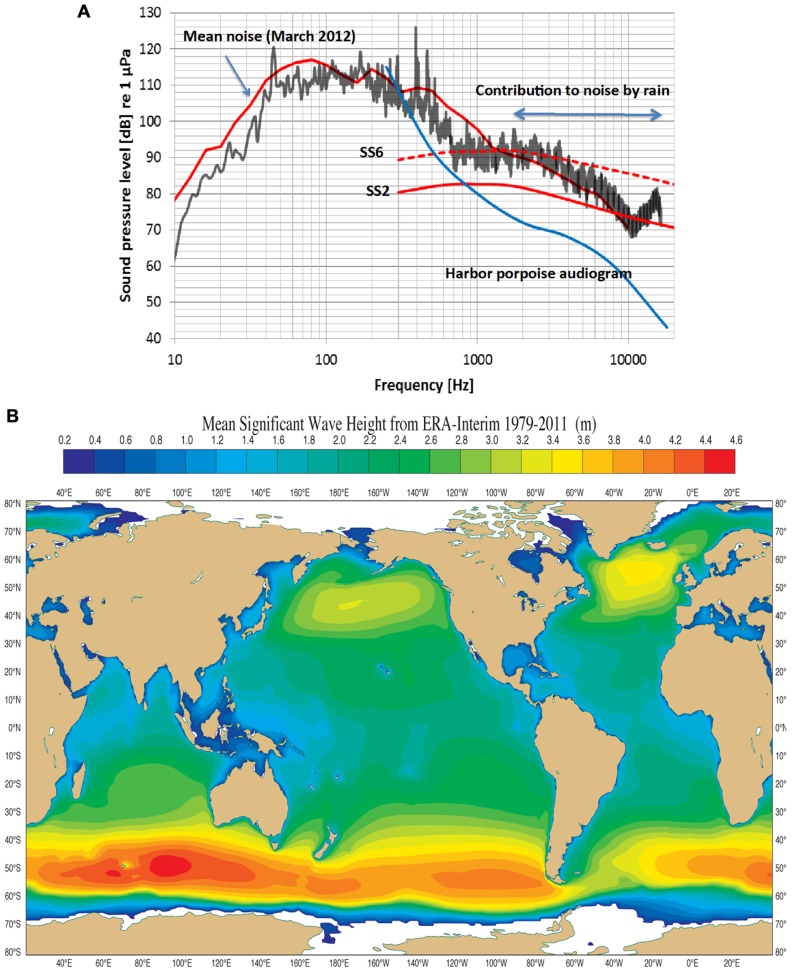
**(A)** An example of noise measurements (third octave sound pressure levels) in the coastal waters of Fehmarn Belt, German Baltic. Sea state 2 and sea state 6 (ss2, ss6) are taken from Wenz, 1962. The mean curve during the month of March 2012 includes ship noise, which contributes mostly below 1 kHz. The black curve is a 20 min measurement of noise during rain (30–40 mm/h) during sea state 2, raising the noise level from 5 to 10 dB. The “contribution to noise by rain” begins to die out above 16 kHz. The blue curve is the lower portion of a harbor porpoise audiogram (Kastelein et al., 2002). Note that the harbor porpoise can hear all sources of noise above 1 kHz. (Courtesy of Dr. Dietrich Wittekind, DW-ShipConsult, Schwentinental, Germany and funded by the German Federal Agency of Nature Conservation). **(B)** The mean significant wave height (H_s_) from 1979 to 2011 in most of the World’s oceans lies between 1 and 3 m corresponding to mean Sea States between 3 and 5. Noise from these Sea States meets thermal noise at about 130 kHz, the biosonar frequency of the harbor porpoise (Dee et al., 2011; and courtesy of Dr. Jean Bidlot, The European Centre for Medium range Weather Forecasts, Reading, UK).

Wenz (1962) is the standard reference for noise in the open-ocean and coastal areas. There are still surprisingly few studies of coastal water acoustics and all but one deal with lower frequencies outside the NBHF echolocation signals used by the harbor porpoise (see for example Wilson et al., 1985; Piggott, 1964). Recently, however, the noise profile in Fehmarn Belt (coastal waters in the German Baltic) was determined for March, 2012 (**Figure 2A**). The mean noise profile (upper red curve) includes natural and anthropogenic sources while the black curve is a 20 min measurement of noise during rain at sea state (SS) 2. The noise levels in **Figure 2A** follow the general trend in that the levels are about 10 dB higher for frequencies above 1 kHz in Fehmarn Belt relative to those in open ocean waters. Rain contributes to high frequency noise and this is maximum at about 15 kHz at a level of about 88 dB re 1 μPa (1/3 octave band). Harbor porpoises are common in the Fehmarn Belt and the hearing threshold (Kastelein et al., 2002) for the lower frequencies of the audiogram is plotted in **Figure 2A**. It is obvious that harbor porpoises can easily hear noise above about 500 Hz. Rain noise is apparently quite irritating since the animals in the Fjord&Bælt facility at Kerteminde begin to swim rapidly, breaking the water surface (“porpoising”) while doing so, for an extended time during rainfall. The same has been observed for several harbor porpoises in facilities in the Netherlands (R. Kastelein, personal communication). This shows that sound outside the frequency range of porpoise biosonar may cause the animal to abort any prevailing behaviors like foraging.

## CLUTTER IN THE COASTAL ENVIRONMENT

In biosonar, we define clutter as unwanted echoes from objects near the target of interest. Odontocetes emit their biosonar in directional beams. The beams are shaped like cones having a width in degrees defined by an arbitrary number of dB down from the central axis of the beam, often -3 or -10 dB (Au, 1993). The further the harbor porpoise is from the target of interest, a fish for example, the greater is the ensonified area. If the porpoise can perceive an echo from the prey then it can also perceive clutter echoes from other objects in the biosonar beam having similar echo strengths, which presents problems for detecting prey.

## ACOUSTIC ADAPTATIONS FOR NOISE AND CLUTTER

Even though the coastal environment offers abundant and varied prey, finding and capturing it presents several challenges for an odontocete. How does it deal with the general increase in noise level of the coastal environment? What about the plethora of uninteresting clutter echoes from for example bottom structures in relatively shallow water? How is the predator avoiding becoming prey to e.g., the killer whale (*Orcinus orca*)?

Almost all echolocating animals use ultrasonic signals. Ultrasound is needed to get echoes from small objects. Harbor porpoise echolocation signals have a wavelength slightly larger than 1 cm and can be used to obtain good echoes from prey items of this or even smaller size, in other words very small fish. The harbor porpoise NBHF signals have a more than 20 dB lower intensity than most other Odontocetes, but the signals are significantly longer in duration. Thus, the returning echoes will have a lower intensity, a narrower bandwidth and a longer duration as compared to signals emitted by most dolphins. A series of narrow-band auditory filters seems to improve the ability for the harbor porpoise to extract an echo from broad-band noise (**Figures 1C**,**D**).

There are basically three ways the harbor porpoise can deal with clutter echoes. One is to reduce the amplitude of its biosonar signals so it can perceive echoes from the target but cannot hear clutter echoes from objects having lower target strengths than that of the target. The harbor porpoise does reduce the amplitude of its biosonar as it approaches a prey item (Atém et al., 2009; Miller, 2010). A second way to reduce clutter echoes is to have a narrow sound beam or better yet to be able to change the width of the beam. Being small, like the harbor porpoise, means that it is difficult to maintain high signal directionality. Directionality is mainly governed by the frequency content of the signal and the size of the transducer. In addition, air sacs, cranial structures, and variations of the speed of sound within the melon help to improve directionality (Au, 1993). Having a high frequency signal (approximately 130 kHz) is an advantage since directionality is proportional to frequency for the same emitter size. Using even higher frequencies to get more directionality would be a disadvantage because of increased sound attenuation. Hearing sensitivity would have to follow suit, but this decreases rapidly above 140 kHz (Kastelein et al., 2002, 2010). So having a narrower sonar beam would improve the echo to clutter ratio, but too narrow a beam would be problematic. Naturally the porpoise can steer the beam by moving its head, like visual gazing (Verfuss et al., 2009; Wisniewska et al., 2012). Being able to adjust the beam width would be a great advantage for clutter rejection, but if it can do this, as some bats can (Jakobsen et al., 2013), is unknown. Thirdly, to reject clutter from objects farther than that of interest, the harbor porpoise could use an auditory temporal window, similar to that found in certain bats (Miller, 1991), which would allow processing of echoes in a restricted range. If the porpoise can do this is unknown.

## SELECTION PRESSURES FOR ADOPTING A NARROW BAND HIGH FREQUENCY BIOSONAR SIGNAL

Killer whales prey upon harbor porpoises and other marine mammals. Killer whale hearing is best at 20 kHz and one animal showed behavioral responses at 120 kHz. By extrapolation, the behavioral hearing threshold near 130 kHz would be about 90 dB re 1 μPa RMS for 2 s tone bursts (Szymanski et al., 1999). This means that a killer whale should be able to hear the biosonar of a harbor porpoise at up to about 0.5 km (assuming spherical spreading loss, a sound absorption of 40 dB/km and a short auditory time constant of the killer whale) since a wild harbor porpoise can have a source level of about 190 dB re 1 μPa pp (Villadsgaard et al., 2007). This could be a cue for killer whales, which are known to take both harbor and Dall’s porpoises (*Phocoenoides dalli*; Matkin et al., 2007). Still, the special characteristics of harbor porpoise biosonar signals certainly make it difficult for killer whales to detect them and may be the selection pressure that drove the harbor porpoise signal to higher frequencies (Andersen and Amundin, 1976; Madsen et al., 2005; Morisaka and Connor, 2007).

There could be another selection pressure driving harbor porpoise biosonar, and that of other odontocetes using NBHF signals, upward into a narrow band of frequencies around 130 kHz. There is a direct relationship between noise level, wind velocity, wave height (H_s_) and SS (Wenz, 1962). In addition, as frequency increases from about 10 kHz and upward so does thermal noise by a factor -15 dB + 20 log *f* where *f* is frequency in kHz (Urick, 1983). SS noise at levels of SS2 to SS4 meet thermal noise at about 130 kHz (Wenz, 1962), forming a minimum of combined SS and thermal noise at a level of about 60 dB re 1 μPa rms (assuming a 4 kHz auditory filter bandwidth of the harbor porpoise (Popov et al., 2006). An analysis of mean significant wave H_s_ in the world’s oceans over 33 years shows wave H_s_ are mostly around 2.4–2.6 m except for smaller areas in the North Pacific and North Atlantic, and the Southern Oceans (**Figure 2B**; Dee et al., 2011). This corresponds to a SS of 4. Measurements in the Mid-Atlantic off Florida and the Pacific off of Baja California gave similar SS values (NASA, 2000). This means that the harbor porpoise listening at 130 kHz cannot hear SS noise below about three because thermal noise dominates, but it can easily hear SS noise of four and above since these dominate when listening at 130 kHz (Kastelein et al., 2002, 2010). If sea states over geological time were at levels 3 and 4 and thermal noise was as it is today then these combined noise sources have a minimum at about 130 kHz. Thus, we hypothesize that the minimum level of sea noise and thermal noise at 130 kHz was a strong selective factor in the evolution of NBHF biosonar in some odontocetes.

Support for the above can be derived from the diversity of species using NBHF biosonar and cranial morphometrics (Galatius et al., 2011). Dall’s porpoise has a substantially larger skull than that of the harbor porpoise; larger by ca. 23% in a comparison of both sexes of Californian *Phocoenoides dalli* and *Phocoena phocoena* from the inner Danish waters. In the same comparison, the skeletal structures surrounding the sound producing apparatus were relatively larger in *Phocoenoides dalli* (Galatius et al., 2011). Thus, judging from skull morphometrics the peak frequencies of Dall’s porpoise biosonar clicks should differ significantly from those of harbor porpoises, but they do not. The peak frequency of both species, and others, is nearly the same at about 130 kHz (Møhl and Andersen, 1973; Au, 1997). Thus, in the near coastal NBHF species, selection has been for a similar size of the sound generating apparatus (phonic lips etc.) that can produce approximately130 kHz biosonar and not for cranial size.

## CONCLUSION

We conclude that over time selective pressure from predation by killer whales may have pushed biosonar up in frequency while the meeting point of SS noise and thermal noise formed a minimum at about 130 kHz providing a convenient end point for narrow band high frequency biosonar. Harbor porpoises can effectively extract echoes from the extra noise in coastal water using their narrow band auditory filters. We propose they also listen with broadband filters to improve temporal resolution.

## Conflict of Interest Statement

The authors declare that the research was conducted in the absence of any commercial or financial relationships that could be construed as a potential conflict of interest.
